# 50 YEARS OF NEWBORN SCREENING FOR CONGENITAL HYPOTHYROIDISM: EVOLUTION OF INSIGHTS IN ETIOLOGY, DIAGNOSIS AND MANAGEMENT: Transient or permanent congenital hypothyroidism: from milestones to current and future perspectives

**DOI:** 10.1530/ETJ-25-0019

**Published:** 2025-08-05

**Authors:** Gaia Vincenzi, Ilenia Teresa Petralia, Marco Abbate, Maria Cristina Vigone

**Affiliations:** IRCCS Ospedale San Raffaele, Department of Paediatrics Milano, Lombardia, Italy

**Keywords:** re-evaluation, gland in situ, congenital hypothyroidism, screening

## Abstract

Primary congenital hypothyroidism (CH) is the most common endocrinopathy of developmental age. In recent years, several studies from different countries have reported a significant increase in CH incidence detected by newborn screening programs, primarily ‘mild’ forms of CH with gland *in situ* (GIS). However, more than one-third of affected children with GIS present transient CH and recover endogenous thyroid function in early childhood, permitting the cessation of levothyroxine treatment by the end of the third year of life. Therefore, in CH patients with GIS, a clinical and biochemical reassessment is needed to determine whether the hypothyroidism is transient or permanent and to search for the underlying causes of the thyroid defect. Despite the presence of consensus guidelines for the management of CH in pediatric age, the screening strategy and management of the disease, especially at re-evaluation, differ significantly between centers and present some points of discussion. The following review summarizes the main pathophysiological mechanisms of transient and permanent forms of CH, also underlining the importance of new genetic tools in order to guarantee each patient the best diagnostic and therapeutic approach.

## Background

Primary congenital hypothyroidism (CH) is the most common endocrinopathy of developmental age. For decades, neonatal screening has allowed early diagnosis and initiation of replacement therapy with levothyroxine (L-T4), which is essential to ensure proper neurocognitive development in affected newborns. Nowadays, the incidence of CH confirmed at birth shows great variability (1:1,000–1:3,700 live births) due to different neonatal screening strategies in different countries (1). The incidence is higher when screening programs include measurement of TSH or both TSH and T4 and consider strategies for newborns at risk of delayed TSH elevation (2, 3, 4, 5, 6, 7). In contrast, a lower incidence has recently been reported in the Netherlands and other countries where the screening program is based on T4 measurement alone (8). In addition, the progressive lowering of the TSH cut-off has been the main cause of the increasing incidence of primary CH (9), as it allows the identification of a greater number of ‘mild’ forms with slight elevation of TSH, mainly but not only with GIS. The increasing incidence of CH is influenced not only by the adopted newborn screening strategy but also by other factors such as the increasing number of newborns at risk of developing CH, such as premature or low birth weight infants, twins, and iodine deficiency during pregnancy.

CH can present with thyroid dysgenesis or with a GIS. While thyroid dysgenesis suggests a permanent form, eutopic gland can account for either permanent or transient forms. In 2011, LaFranchi *et al.* suggested that permanent primary CH (PCH) due to thyroid dysgenesis could account for approximately 85% of cases, with a prevalence of ectopic gland followed by thyroid aplasia or hypoplasia (8). In 2015, Olivieri *et al.* re-evaluated the incidence trend of PCH and its different etiologies. In 1995–2008, a dramatic increase (225%) in PCH with normal or hypoplastic thyroid was shown, whereas the incidence of PCH due to thyroid dysgenesis (agenesis, ectopy, hemiagenesis) presented only a slight increase (8.0%). In summary, CH due to defects in thyroid embryogenesis accounted for approximately 58% of cases, while the remaining 42% could be related to defects in thyroid hormone synthesis (10). Over time, as the incidence of ‘mild’ forms of CH has been increasing, so has the incidence of transient forms (11). Therefore, in patients with CH due to a GIS, a biochemical re-evaluation is needed to determine whether the hypothyroidism is transient or permanent and to identify the underlying causes in order to evaluate the best possible therapeutic and follow-up strategy. In 2013, our center analyzed the clinical and biochemical features and the outcomes of 84 patients with CH and eutopic thyroid gland (GIS) who systematically underwent re-evaluation after the age of 3 years. Remarkably, more than one-third of patients (38.1%) showed a complete normalization of thyroid function after treatment withdrawal, confirmed after at least 1 year of follow-up (11). This prevalence of transient congenital hypothyroidism (TCH) is similar to that reported in previous studies (12) and highlights the importance of diagnostic re-evaluation to avoid unnecessary treatment later in life. Finally, the idea of TCH and PCH as a continuum of the same disease rather than two separate entities is becoming increasingly interesting: some individuals with only partially impaired thyroid hormone biosynthesis due to specific genetic defects, for which alternative pathways can functionally compensate, may be able to maintain euthyroidism when thyroid hormone requirements are at basal levels, although L-T4 supplementation may be required when metabolic demand is increased, for example during the neonatal period, adolescence, and pregnancy (13).

### Prematurity

The production of thyroid hormones can vary considerably in the first weeks of life, and their reference range varies according to gestational age and also postnatal age (14). Preterm newborns present a typical thyroid function pattern: at birth, as in full-term infants, an increase in TSH levels is observed in the first hours postpartum. However, in premature infants, the peak is more modest, oscillating between 30 and 50 mIU/L. Contrary to what is observed in full-term newborns, in newborns born between the 23rd and 27th weeks of gestation, serum total T4 levels decrease in the first week of life; in newborns born between the 28th and 30th weeks of gestation, serum T4 presents within range, while only babies born after 30 weeks of gestation show an increase in total T4 in the first week. Serum levels of fT4, the active form of the hormone, behave similarly. T3 concentrations are proportionally even lower, especially in more premature newborns (14, 15, 16). TCH is more common in preterm newborns, with an incidence of 1:1,114 (17).

CH may develop as a result of maternal exposure to antithyroid medications or fetal exposure to TSH receptor-blocking antibodies (9, 18). Moreover, the use of iodine-based skin disinfectants on premature infants can inhibit thyroxine production, resulting in transient hypothyroidism (19, 20). Finally, untreated maternal hypothyroidism might lead to low fetal levels of thyroxine as well (19, 20).

Studies carried out in our center have identified a higher incidence of transient forms of CH in premature patients (52.4%) than in term babies, and the analysis of the etiology of CH in our premature population highlights the high prevalence of CH with a eutopic thyroid (87.5%) (21). These data underline the importance of diagnostic reassessment later in life in order to define transient and permanent forms.

Therefore, it is also of crucial importance to identify phenomena such as hypothyroxinemia of prematurity or delayed elevation of TSH, which differ from the typical forms of CH (16). In addition, preterm infants with clinical features such as twin birth, IVF, SGA-associated malformations, mild neonatal respiratory distress requiring respiratory support, and severe postnatal complications of prematurity (anemia, sepsis, intraventricular hemorrhage, retinopathy, transfusion, and administration of drugs such as dopamine and steroids) may influence thyroid function in the neonatal period (21, 22).

Newborn screening plays a vital role in detecting CH, especially in preterm infants. Caiulo *et al.* strongly support both the use of second screening and a lower cut-off for second screening: preterm infants less than 34 weeks’ gestation are more likely to have blood TSH (b-TSH) levels between 5 and 9.9 mIU/L than 10 mIU/L or higher. These infants are therefore at risk of being missed if a second screening cut-off of 5 mIU/L is not used (6).

### Iodine deficiency or excess

Iodine is a micronutrient required for the production of thyroid hormones, which regulate metabolism, growth, and neurodevelopment, and it is well known that in areas of severe and chronic iodine deficiency, hypothyroidism can occur in both mothers and fetuses from the early stages of pregnancy, leading to irreversible brain damage, mental retardation, and neurological abnormalities (23). The mechanism by which the thyroid adapts to inadequate iodine supply is by increasing iodide uptake and subsequent steps in intrathyroidal iodine metabolism, leading to preferential synthesis and secretion of triiodothyronine (T3). These mechanisms are triggered and maintained by increased secretion of TSH, which is ultimately responsible for the development of goiter (24). Neonates appear to be hypersensitive to the effects of iodine deficiency (25). This characteristic is explained by a particularly low iodine content of the neonatal thyroid and an accelerated turnover rate of their intrathyroidal iodine reserves. The turnover rate is 1% in adults and 17% in neonates under iodine repletion conditions, but 62 and 125% in moderate and severe iodine deficiency conditions, respectively (25). Several studies have reported the causal role of iodine deficiency in the pathogenesis of TCH, and increased incidence of TCH in iodine-deficient areas has been reported worldwide (24, 25, 26). Although global improvements in iodine status over the past 25 years have resulted in major health and economic benefits, particularly in low- and middle-income countries, this strategy has not been adopted in many regions of the world, including the USA and much of Europe. Even in some regions where salt iodization programs are well established, pregnant women, who have higher iodine requirements, may have inadequate iodine intake. The World Health Organization (WHO) provides guidelines for iodine supplementation based on household consumption of iodized salt (27). In regions where less than 90% of households consume iodized salt and the median urinary iodine concentration is less than 100 μg/L, iodine supplementation in the form of potassium iodide is recommended, with a target intake of 250 μg/day. Ideally, iodine supplementation should be started at least 3 months before conception to ensure that maternal thyroid iodine stores are sufficient for the entire pregnancy (27, 28).

However, on the other hand, safe upper limits for iodine intake in pregnant women, lactating women, and infants have not been comprehensively defined (29). Excessive iodine intake can also have a negative effect on thyroid function. Normally, high doses of iodine cause a temporary shutdown (from a few days to about 2 weeks) of thyroid hormone production (the acute Wolff–Chaikoff effect). It is proposed that iodopeptide(s) are formed that temporarily inhibit thyroid peroxidase (TPO) mRNA and protein synthesis and, therefore, thyroglobulin iodination. The Wolff–Chaikoff effect is an effective means of rejecting the large amounts of iodide and, therefore, preventing the thyroid from synthesizing large amounts of thyroid hormones (30). Continuous exposure to high levels of iodine causes downregulation of the sodium iodide symporter (NIS), which transports iodine into the thyroid gland, allowing thyroid hormone production to continue; this is known as escape from the acute Wolff–Chaikoff effect (29). Failure of this effect can lead to iodine-induced hyperthyroidism or hypothyroidism, especially in people with pre-existing thyroid disease. Susceptible patients with identified risk factors may be at increased risk of failing to adapt to the acute Wolff–Chaikoff effect. Vulnerable patients include those with autoimmune thyroid disease; a history of surgery, 131I, or antithyroid drug therapy for Graves’ disease; subacute thyroiditis; postpartum thyroiditis; amiodarone-induced type 2 thyrotoxicosis (AIT); hemithyroidectomy; IFNα therapy; and concomitant use of potential goitrogens such as lithium (29, 30, 31). Failure to escape the acute Wolff–Chaikoff effect may also be more likely during fetal development, a period when the hypothalamic–pituitary–thyroid axis is immature, and during neonatal life. The fetus is particularly vulnerable to hypothyroidism because the ability to fully escape this effect does not develop until about 36 weeks of gestation. An example: maternal or neonatal administration of amiodarone as an antiarrhythmic drug may cause transient hypothyroidism in the fetus or neonate. Amiodarone contains a high iodine load, which leads to hypothyroidism by interfering with thyroid hormone biosynthesis through the Wolff–Chaikoff effect. Transient hypothyroidism also occurs when iodine antiseptics are used in mothers or after exposure to iodinated contrast media. The effects may be related to the type and duration of exposure (32). Neonatal exposure to iodine may occur particularly in preterm infants. In addition, neonates with congenital heart disease are particularly vulnerable because they may be exposed to several sources of excess iodine simultaneously, including large intravenous contrast loads during cardiac catheterization and topical iodine-containing antiseptics and dressings after surgical procedures (33, 34).

### Transfer of maternal blocking antibodies

TSH receptor antibodies (TRAbs), immunoglobulins of the gamma subtype (IgG) directed against the thyrotropin receptor (35), can cross the placenta from about 16 weeks of gestation. TRAbs could play a pathogenic role in the development of hypothyroidism in both mother and baby by blocking the effects of TSH (36, 37, 38). The incidence of CH due to TSH receptor-blocking antibodies (TBAbs) is probably underestimated because TBAbs are rarely evaluated in pregnant women with hypothyroidism. TBAbs are found in both women with Graves’ disease and Hashimoto’s thyroiditis (39). A high titer of TBAbs can lead to profound hypothyroidism in the newborn, with thyroid hormone levels at diagnosis similar to athyreosis. In these patients, it is essential to perform careful ultrasound and scintigraphic imaging at diagnosis, as well as measuring serum thyroglobulin (38). This effect of maternal TRAbs can last up to 3–4 months after birth as maternal antibody levels fall; hypothyroidism is therefore typically transient. Perhaps the effect of TRAbs could also be related to persistent forms: Evans *et al.* described persistent mild thyroid dysfunction with a small thyroid lobe, probably related to the inhibition of normal TSH receptor signaling during the antenatal and neonatal periods (37). Finally, a careful maternal anamnestic evaluation, as well as the evaluation of maternal thyroid function and antibody status, plays a fundamental role in the early identification of hypothyroidism caused by TRAbs in order to avoid unnecessary prolonged treatment and monitor future pregnancies for risk of recurrence of this condition (13).

### Assay interferences

Analytical interference in thyroid function tests (TFTs) can lead to diagnostic delays, unnecessary testing, and inappropriate treatment (40). The prevalence of analytical interference leading to erroneous immunoassay results is 0.4% (41). The most common sources of interference in TSH assays are endogenous antibodies, the most common of which are human anti-animal antibodies, rheumatoid factors, and autoantibodies to the analyte (in this case, TSH). The latter are responsible for the so-called ‘macro-TSH’, which is a large (>150 kDa) circulating form of TSH composed of monomeric TSH (28 kDa) complexed with anti-TSH autoantibodies. Macro-TSH is a bioinactive macromolecule that is not readily filtered by the kidney and accumulates in the serum. It is found in 0.5–1.6% of samples referred for hypothyroidism (42, 43). Transplacental passage of maternal interfering immunoglobulins usually causes an elevated TSH with normal FT4 and FT3 in a clinically euthyroid newborn, and a similar thyroid pattern is present in the mother. It is therefore crucial to measure maternal thyroid function in this context, as an elevated maternal TSH with normal FT4 and FT3 supports the diagnosis, and confirmation of an artifactually elevated TSH will avoid inappropriate levothyroxine treatment in a clinically euthyroid baby. Interference in the TSH assay resolves at around 8 months of age, in accordance with a normal rate of maternal IgG clearance, while maternal TSH levels remain high (13, 44).

### Drugs

It is known from the literature that various drugs used during pregnancy and/or the neonatal period can interfere with maternal and/or fetal thyroid function. As previously discussed, the use of amiodarone antiarrhythmic therapy during pregnancy results in excessive fetal exposure to iodine. Iodine overload may be responsible for persistent inhibition of fetal thyroid function, which can lead to hypothyroidism and goiter (45).

In addition, antithyroid drugs such as propylthiouracil (PTU) or methimazole (MTZ) can cross the placenta and cause a decrease in thyroid hormone production in the fetus. In particular, fetal free thyroxine (fT4) levels tend to be lower than maternal fT4 levels when mothers require ATD treatment before delivery (46), and the lowest possible dose of ATD should be used whenever possible (27). PTU and MTZ carry a similar risk of causing fetal hypothyroidism or neonatal TCH. Several studies have reported conflicting results regarding the dose–response relationship between maternal ATD doses and neonatal thyroid function. In particular, cord serum PTU concentrations may also be higher than concurrently obtained maternal serum PTU concentrations, suggesting slower PTU clearance in the fetus (47). In general, clinically evident fetal hypothyroidism is rare at low doses of PTU (≤50 mg daily), and a recent study showed no significant differences between maternal and cord FT4 levels at this dose (48). Both methimazole and PTU are rapidly cleared from the fetal circulation; therefore, neonatal transient hypothyroidism due to ATDs usually resolves within a few days and may not trigger the neonatal diagnostic screening program (49).

The use of dopamine in neonates is also known to cause postnatal thyroid dysfunction. In neonates, particularly premature neonates, dopamine is widely used as a supportive inotropic agent. In fact, it is known that dopamine infusion can induce suppression of neonatal TSH secretion, with an immediate increase in TSH levels after cessation (50), and an association between dopamine infusion and TCH in preterm infants has been demonstrated (51). It should be emphasized that the ability of dopamine to suppress thyroid-stimulating hormone may prevent early diagnosis of CH (52, 53). Therefore, some studies suggest that all newborns should be tested for TSH and thyroxine levels at the time of primary screening, or that thyroid hormones should be re-evaluated after discontinuing dopamine in patients treated with this inotrope (53).

### Genetics

New molecular biology technologies, particularly next-generation sequencing (NGS), which allow the simultaneous analysis of multiple genes, have confirmed the role of oligogenic inheritance in human CH. In particular, Persani *et al.* showed that targeted sequencing analysis of 11 CH-associated genes in 177 Italian patients with different CH subtypes revealed the presence of a likely pathogenic variant in more than one gene in 25% of cases, associated with both eutopic CH (GIS) and thyroid dysgenesis (54, 55).

In France, patients with CH with the gland *in situ* had genetic alterations in NGS that explained thyroid dysfunction in 42% of cases, a percentage that increased to 70% in patients with screening TSH ≥80 mIU/L or FT4 values ≤5 pmol/L (56).

Many genes associated with CH and GIS have been described in the literature: defects in iodine uptake by thyrocytes (NIS/SLC5A5 mutations), partial or total iodine organification defects (P/TIOD) (mutations in the *TPO*, *DUOX2*, *DUOXA2*, and *PENDRIN* (*SLC26A4*) genes), defects in the synthesis, storage, or release of TG, or IYD defects (*DEHAL1*) (57). They all have a recessive autosomal mode of transmission and a variable phenotype (transient, mild, or severe hypothyroidism).

The most common mutations in patients with GIS have been found in *DUOX2* and in the TSH receptor gene (*TSHR*). Several studies from different countries have strongly implicated *DUOX2* and *DUOXA2* in the etiology of transient CH (13, 58, 59); however, three or more *DUOX2* pathogenic variants are mostly associated with permanent forms (60). In contrast, even mild defects in other genes involved either in thyroid hormone synthesis or thyroid development usually cause permanent CH. This may reflect the fact that, unlike *DUOX2*, there are no alternative pathways to compensate for the specific defect in other mild dyshormonogenesis, for example due to TG or TPO mutations (13). Mutations in the TSH receptor deserve a separate discussion. Although compensated hypothyroidism can occur with *TSHR* mutations, this is a permanent rather than a transient condition in which upregulation of TSH synthesis and possible ‘resetting’ of the hypothalamic–pituitary–thyroid axis maintains TSH elevation despite normal pituitary sensitivity to circulating thyroid hormones (61, 62). Resistance to thyrotropin (TSH) caused by monoallelic or biallelic *TSHR* variants has a special place in the etiology of primary CH, with phenotypes ranging from mild persistent hyperthyrotropinemia (PHT) with normal gland size to severe permanent CH (PCH) with orthotopic hypoplasia (61, 63, 64, 65).

Genetic testing for CH (before, during, or after reassessment) is an important tool to support a personalized approach for the management of CH. However, genetic testing is currently reserved for some selected cases, such as permanent hypothyroidism with gland *in situ* and family history of thyroid disease, presence of associated malformations, or chromosomopathies (66). In addition, the use of NGS is still limited to a few selected laboratories.

## The re-evaluation

### When

According to the latest European guidelines, diagnostic re-evaluation (66) is indicated between 2 and 3 years of age in patients with a GIS, or in those in whom a definitive diagnosis of permanent CH has not been made in the first weeks or months of life. An early accurate diagnosis of permanent forms – in most cases achieved by dual imaging (ultrasound + scintigraphy) – avoids the need for further diagnostic testing and reassessment of the cause at a later stage: if thyroid scintigraphy with Tc 99 is not performed at diagnosis in these cases, diagnostic reassessment with thyroid scintigraphy is essential. Therefore, in cases of gland dysgenesis confirmed by scintigraphy, re-evaluation will not be necessary. Furthermore, a re-evaluation should also be carried out in suspected isolated central CH.

Anticipation of suspension at 12–24 months of age can be performed in children of mothers with autoimmune thyroid disease, in patients who have not required an increase in therapeutic dose during the first year of life, or in cases with a history of exposure to excess iodine.

Furthermore, early reassessment at 6 months of age is possible in patients without a diagnosis of permanent CH and with GIS, which requires a dose of L-T4 <3.2 μg/kg per day at 6 months of age (or even 2.5 μg/kg/day at 12 months of age) in patients without a history of familial CH (66, 67).

According to our daily clinical practice, we suggest performing a re-evaluation at 24 months of age in patients who require a dose of L-T4 <2.5 μg/kg per day. In patients who require a dose of L-T4 >2.5 μg/day at 36 months of age, we suggest performing a genetic test (NGS), and then proceeding with the re-evaluation, except in the case of mutations associated with permanent forms, presence of a homozygous mutation in the TG or TPO gene, or compound heterozygosity of TG and TPO.

### How

For accurate re-evaluation, L-T4 treatment should be stopped gradually over a period of 4–6 weeks or just suspended, followed by evaluation after 4 weeks of full TFT. If not possible, at least FT4 and TSH should be measured (66). In cases of severe CH at diagnosis, thyroid tests should be performed after 15–20 days. At the time of re-evaluation, a complete reassessment consists of TFTs (TSH, FT3, and FT4), the dosage of thyroglobulin, and autoantibodies (TGAb, TPOAb, TRAbs). The measurement of thyroid antibodies allows the exclusion of any possible thyroiditis onset. In addition, thyroid ultrasound is recommended, and 123I scintigraphy + perchlorate test only in certain cases, in order to detect iodine uptake defects. Specifically, a 10% reduction in 123I uptake 2 h after oral administration of sodium perchlorate is considered positive for an iodide incorporation defect. Patients with an iodide release of 10–90% are considered to have a partial iodide organizing defect, whereas patients with an iodide release of more than 90% are considered to have a total iodide organizing defect (TIOD) (68). Thus, before the availability of genetic panel testing, the perchlorate discharge test, together with the measurement of serum thyroglobulin concentration, represented a diagnostic tool that helps define the picture of patients with permanent CH with GIS (69). Scintigraphy with I123 and perchlorate testing has lost some of its relevance in recent years, as genetic analysis with NGS has become available and increasingly widespread, allowing a better characterization of the clinical picture.

### Results

TSH within limits with normal FT4: CH is considered to be transient, and thyroid function monitoring is required after a few months.

TSH above the upper limit of the reference range but <10 mIU/L (primary CH) or fT4 just above the lower limit of the reference interval (central CH): continue withdrawal and retest in another 3–4 weeks (66). It is crucial to closely monitor TSH levels in the first months after treatment withdrawal: slightly elevated TSH levels found immediately after withdrawal may both normalize and increase over time, just as normal TSH levels may increase immediately or several months after treatment withdrawal. Unfortunately, there is no consensus in the management of patients with normal FT4 and mild hypothyroidism (TSH 5–10 mIU/L) regarding whether to restart L-T4 therapy, and the approach still varies from center to center.

TSH ≥10 mIU/L and/or FT4 below the lower reference limit: primary hypothyroidism is confirmed; thyroid imaging and, if possible, genetic testing should be considered. L-T4 therapy should be restarted (66).

Conversely, if TSH values are slightly >10 mIU/L with adequate FT4 values, we suggest considering the possibility of a close dosage (15–20 days) before restarting treatment in order to assess the TSH trend. In fact, in our clinical practice, we have often observed transient increases in TSH values immediately after the suspension of treatment, with subsequent normalization at later check-ups. Moreover, we cannot exclude the possibility that, in those patients requiring L-T4 treatment after the re-evaluation, a second attempt at therapy withdrawal at the end of puberty might provide new information about the progression of thyroid dysfunction.

If fT4 is below the lower limit of the reference range, along with a mildly elevated or normal TSH, and central CH is still suspected, all anterior pituitary axes should be tested, and genetic analysis should be performed (66).

**Figure 1:**
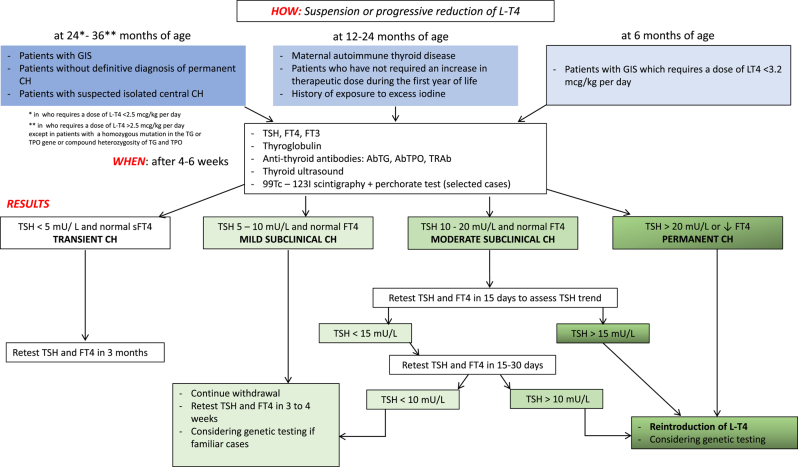
Diagnostic re-evaluation flow chart in patient with GIS. L-T4, levothyroxine; CH, congenital hypothyroidism; GIS, *in situ* gland; AbTG, thyroglobulin antibodies; AbTPO, thyroid peroxidase antibodies; TRAb, TSH receptor antibodies; 99Tc, Technetium 99; 123I, Iodine 123.

Figure 1 illustrates the diagnostic re-evaluation flow chart in patients with GIS.

## Predictors of outcome

At the time of diagnosis, it is not possible to distinguish between permanent and transient forms of GIS. The severity of neonatal hypothyroidism depends on the underlying etiology, which may be genetic, environmental, or a combination of both (13). Thyroid hormone deficiency in the neonatal period may be profound, with severe deficiency of FT4 and significantly elevated TSH concentrations, also in children with transient CH at the re-evaluation. On the other hand, mild CH at diagnosis could also imply permanent forms requiring L-T4 therapy for the entire life.

Various authors have tried to identify the risk factors associated with transient/permanent forms, with conflicting results. Male sex (70, 71), non-white ethnicity (72), low birth weight (73, 74, 75), neonatal illness, and lower severity at diagnosis (70, 73, 74, 76, 77, 78, 79) seem to be more frequently associated with transient forms. Prematurity (10, 11, 80), concomitant congenital abnormalities (77), a family history of thyroid disease (4, 11), thyroid hypoplasia (11, 81), and higher L-T4 dose requirement seem to be associated with permanent forms (4, 5, 74). Many studies have tried to find a therapy cut-off at 6, 12, or 24 months capable of best distinguishing patients with permanent from transient CH (4, 73, 74, 78): the cut-off of 3.2 μg/kg/day of L-T4 dose at 6 months is considered in the literature to be a good predictor of CH evolution (67).

### Conclusions and future perspectives

The importance of neonatal screening for CH is nowadays worldwide recognized: identifying affected newborns permits prompt start of L-T4 therapy in order to prevent the irreversible neurodevelopmental delay associated with CH in the pre-screening era and guarantee an optimal developmental outcome. Indeed, international guidelines recommend starting L-T4 treatment as soon as possible and not later than 2 weeks after birth in the presence of serum TSH >20 mIU/L at confirmatory tests. An increased incidence of ‘mild’ forms of CH (TSH: 6–20 mIU/L) has been reported in recent years due to the use of a multiple-step screening program, the lowering of the TSH threshold, as well as different cut-offs used between the first and second screening, and the increasing number of newborns at risk, such as premature infants and twins. Therefore, it is not unusual to bump into clinically euthyroid newborns beyond the age of 21 days with serum TSH concentrations between 6 and 20 mIU/L and fT4 concentration within the age-specific reference interval. In these cases, the common query among pediatric endocrinologists is whether to start L-T4 therapy immediately or to wait, re-test, and later re-evaluate the need for treatment. A careful maternal and perinatal anamnesis is of fundamental importance in this choice, which sometimes permits avoidance of unnecessary treatment. On the other hand, a thyroid ultrasound would be advisable also in these patients since not infrequently thyroid dysgenesis could present with mild thyroid dysfunction. Thyroid ultrasound should be performed by an expert radiologist in ultrasound, as in many cases the thyroid is not described, though present in scintigraphy, or initially described and not found in later investigation. The increasing incidence of mild GIS, the decision whether to start or not L-T4 therapy, and the relative implications on neurodevelopmental development are current matters of debate (13). Lain *et al.* showed that children with slightly elevated newborn TSH results, but below the levels indicated for L-T4 treatment in the Australian program, presented poorer performances than children without CH or with CH in L-T4 therapy (82). On the other hand, according to Alm *et al.*, neurodevelopmental outcomes were similar between children with slight hyperthyrotropinemia and unaffected controls (83). Thus, further studies are needed in order to detect the neurodevelopmental effect of treated versus untreated neonatal hyperthyrotropinemia.

Diagnostic re-evaluation is essential in all patients with GIS in whom an etiological diagnosis has not been made in the neonatal period or in whom no cause of permanent CH has been found, with the aim of early discontinuation of treatment in patients with transient thyroid dysfunction. However, the management of CH, particularly at re-evaluation, varies widely between centers, either in timing or methods, despite the presence of consensus guidelines. According to current European Guidelines, re-evaluation is suggested in all patients affected by CH with gland *in situ* between 2 and 3 years of life, a period in which myelination of the nervous system is completed (66). Carrying out a re-evaluation before 2 years of life could therefore be considered a ‘risky’ choice after all the attention taken in promptly starting L-T4 therapy. However, a personalized approach at the time of re-evaluation based on possible predictor factors of transient versus permanent form of CH would be desirable nowadays and in the coming years: approximately 30 percent of infants born at full term and 50 percent of infants born prematurely will present with a picture of TCH. Although it is well known that data at diagnosis do not allow a certain differentiation between transient and permanent forms, several studies have tried to identify potential predictive factors, but they are inconclusive. On the other hand, a personalized approach should be reserved for patients with a GIS requiring high doses of L-T4 in the first 6–12 months of life: a precocious genetic assessment with NGS panels could justify avoiding unnecessary therapy suspension in cases of genetically confirmed permanent forms, such as those supported by mutations in TPO or TG gene in homozygosity or compound double heterozygosity. Genetic consulting is suggested not only in cases of persistent GIS but also in cases of syndromic forms and familial cases; furthermore, we strongly suggest performing NGS also in those cases characterized by mild hyperthyrotropinemia with normal FT4. Detecting defects in particular genes allows the pediatric endocrinologist to establish a personalized follow-up and therapeutic strategy for each patient. For example, clinically well patients with slightly elevated TSH values (5–10 mIU/L) and normal FT4 with heterozygous variants in the TSHR gene might not require L-T4 therapy, while patients with the same thyroid function pattern but with heterozygous TG variants could benefit from L-T4 therapy due to the increased risk of thyroid nodules and cancer described in the literature (84, 85). These patients could also benefit from a tighter ultrasound follow-up. Eventually, the possibility of detecting alteration in genes involved in iodine organification has made scintigraphy with I 123 + perchlorate test lose its clinical relevance in the re-evaluation of GIS. Finally, even if pathogenic variants in *DUOX2* or *DUOXA2* turn out to be often associated with subclinical hypothyroidism (SCH) or, it is important to note that these patients should be offered long-term monitoring for recurrent thyroid dysfunction, especially during adolescence and pregnancy, when metabolic demands are increased (13).

In conclusion, the early identification of patients with transient versus permanent CH has a variety of important implications related not only to decisions regarding the single patient’s follow-up and therapeutic approach. The possible effect of under-treatment is usually an important matter of concern when approaching CH; however, potential effects of chronic overtreatment should also be considered. Finally, other aspects, such as parental anxiety and health care expenses, should also be evaluated. Further studies are needed to better define the framework and assess the interactions between genetics, epigenetics, and environmental factors in defining transient/permanent CH.

## Declaration of interest

The authors declare that there is no conflict of interest that could be perceived as prejudicing the impartiality of the work reported.

## Funding

This review did not receive any specific grant from any funding agency in the public, commercial, or not-for-profit sector.
